# Secondary Processes Dominate the Quiescent, Spontaneous Aggregation of α-Synuclein at Physiological pH with Sodium Salts

**DOI:** 10.1021/acschemneuro.3c00282

**Published:** 2023-08-14

**Authors:** Robert I. Horne, Michael A. Metrick, Wing Man, Dillon J. Rinauro, Z. Faidon Brotzakis, Sean Chia, Georg Meisl, Michele Vendruscolo

**Affiliations:** † Centre for Misfolding Diseases, Yusuf Hamied Department of Chemistry, University of Cambridge, Cambridge CB2 1EW, United Kingdom; ‡ College of Medicine, University of Illinois at Chicago, Chicago, Illinois 60612, United States; § Bioprocessing Technology Institute, Agency of Science, Technology and Research (A*STAR), 138668, Singapore

**Keywords:** Parkinson’s disease, protein misfolding, spontaneous α-synuclein aggregation, kinetic mechanisms

## Abstract

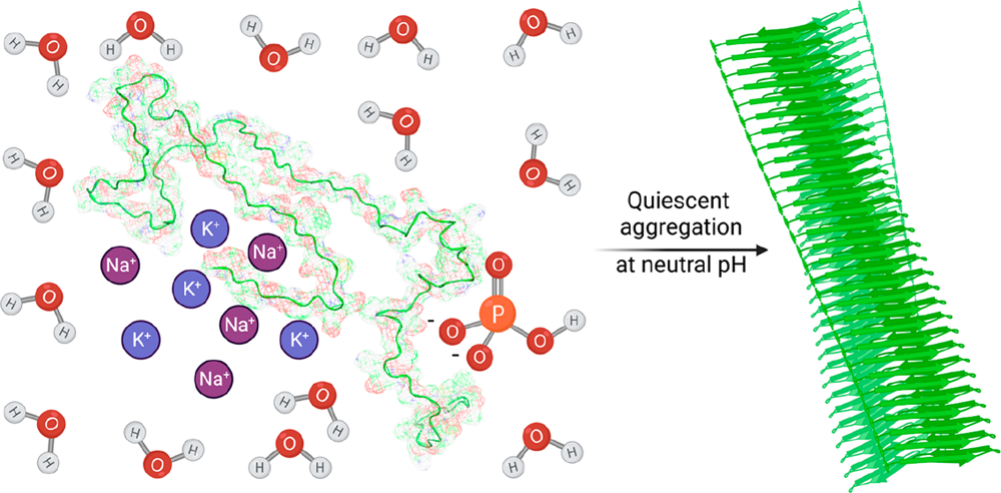

The accurate recapitulation in an in vitro assay of the aggregation process of α-synuclein in Parkinson’s disease has been a significant challenge. As α-synuclein does not aggregate spontaneously in most currently used in vitro assays, primary nucleation is triggered by the presence of surfaces such as lipid membranes or interfaces created by shaking, to achieve aggregation on accessible time scales. In addition, secondary nucleation is typically only observed by lowering the pH below 5.8. Here we investigated assay conditions that enables spontaneous primary nucleation and secondary nucleation at pH 7.4. Using 400 mM sodium phosphate, we observed quiescent spontaneous aggregation of α-synuclein and established that this aggregation is dominated by secondary processes. Furthermore, the presence of potassium ions enhanced the reproducibility of quiescent α-synuclein aggregation. This work provides a framework for the study of spontaneous α-synuclein aggregation at physiological pH.

## Introduction

Alzheimer’s disease (AD) and Parkinson’s disease (PD) are neurodegenerative conditions that are increasingly common in our aging societies^1^ and are still largely incurable. The process of protein misfolding and aggregation, which results in the formation of amyloid deposits, is a hallmark of these diseases and may represent a therapeutic target.^2−4^ The recent approval of lecanemab,^5^ an antibody that slows the cognitive decline in AD by targeting the aggregation of Aβ, which is the main component of amyloid deposits, has strengthened confidence in the amyloid hypothesis.^2,6^ As a consequence, there has been a renewed interest in therapeutic routes for PD based on the targeting of the aggregation of α-synuclein,^7−10^ which is a protein found in Lewy bodies.^11^ These developments are timely, as no disease-modifying drugs have yet been approved for PD.^12^

The rational development of protein aggregation inhibitors can be facilitated by the verification of their mechanism of action through in vitro aggregation assays.^13,14^ Protein aggregation takes place through a complex process that involves a combination of intertwined microscopic steps, including primary nucleation, elongation, and secondary nucleation.^15−17^ Therapeutic candidates can exhibit vast differences in potency depending on which microscopic steps they inhibit.^14,18^ This aspect has been illustrated for AD by the clinical trials of aducanumab and gantenerumab, two antibodies targeting Aβ aggregates for removal. The approval of aducanumab and the failure of gantenerumab correlate with the different mechanisms of action of these two antibodies, as aducanumab mainly inhibits secondary nucleation, whereas gantenerumab mainly inhibits elongation.^19^

For PD, the aggregation process of α-synuclein (αS) has been investigated through methods similar to those developed for Aβ in AD.^20−22^ Observing spontaneous αS aggregation in vitro, however, has been challenging. αS aggregation can be promoted by lipid membranes^20−22^ or by shaking, which introduces air–water interfaces,^23,24^ and is used in diagnostic assays based on biosamples.^25^ The spontaneous aggregation of αS has been recently reported within liquid condensates formed by liquid–liquid phase separation.^26,200^ In these condensates, the concentration of αS reaches millimolar levels, thereby dramatically enhancing the speed of the aggregation process. However, the quest remains open for assays under conditions where spontaneous αS aggregation can be observed in the absence of condensation.

In this work, we report an assay for the spontaneous, quiescent aggregation of N-terminal acetylated αS at pH 7.4 in the absence of lipid membranes. We build upon previous work toward accessing aggregation at physiological pH, where gentle agitation was used (30 rpm for 2–3 weeks).^27^ Here we enable aggregation over a shorter time scale (1–2 days) under quiescent conditions. We established this assay through an optimization of assay conditions focusing on the presence of anions and counter cations.^28^ Given that polyanionic cofactors are likely involved in the core fibril structures of αS fibrils of multiple systems atrophy (MSA) and PD,^29^ we investigated the quiescent aggregation of αS in the presence of sulfate, phosphate, and citrate sodium salts. Such salts induced the quiescent aggregation of αS at physiological pH, where the half-time (*t*_1/2_) of the process, which is indicative of the assay speed, was accelerated and showed good reproducibility. By conducting serial dilutions of initial monomer concentration under different seeding conditions, we found that the kinetics of aggregation followed the behavior expected for a system dominated by secondary processes, with a weak dependence on monomer concentration.

The assay that we report can serve to supplement existing αS assays, providing a battery of tests to verify the mechanism of action of small molecules that inhibit αS aggregation at physiological pH. Further work may identify specific conditions, especially cofactors of αS, that would allow for aggregation to occur at physiological pH with cellular ionic concentrations, in the absence of shaking or seeding surfaces.

## Results and Discussion

### Spontaneous, Quiescent Aggregation of αS in High Salt Concentrations

Methods in this work were designed to keep the ionic strength constant such that the character of the ions could be investigated, independent from the effect of the concentration of ions in solution. Assays that use brain-derived seeds designed to achieve the faithful strain propagation of MSA and PD aggregates via templating recombinant αS have focused on the optimized use of strongly hydrated anions such as sodium citrate.^30,31^ Ensuring retention of the original fibril polymorph during aggregation has proved challenging and requires precise tuning of the aggregation conditions.^32^ Thus, we first sought to accelerate the extremely slow kinetics of quiescent aggregation of αS at pH 7.4, which previously required beads and other nonphysiological cofactors, in the presence of sodium salts at 1 M ionic strength. The quiescent aggregation of αS was enhanced by the presence of divalent anions including SO_4_^2–^ and HPO_4_^2^ (Figure S1). Anions of sodium salts are organized in descending hydration order from left to right. These initial experiments suggested that ionic character, independent of strength and hydration status in the Hofmeister series, is an important factor in quiescent αS aggregation.

We then focused our optimization efforts on sodium phosphate (NaPi), as its reduction of αS kinetic lag times was most pronounced. It was also suggested from the cryo-EM structures of αS fibrils from MSA, PD, and dementia with Lewy bodies (DLB) patient brain extracts that the interfilament interface may be packed with divalent anions such as pyrophosphate.^29,33^ We conducted further optimization experiments and investigated the role of potassium ions in quiescent αS aggregation, as potassium is the main intracellular cation and could thus be a physiological coaggregator alongside αS oligomers and fibrils. Figure S2 shows the effects of increasing NaPi (left to right) in the absence (Figure S2A) or presence (Figure S2B) of 150 mM KCl on quiescent αS aggregation. A dependence of the lag time on the NaPi concentration is shown as increasing NaPi accelerates the quiescent reaction. At an identical concentration of NaPi, the addition of 150 mM KCl accelerates such lag times as much as 3-fold in the case of 400 mM NaPi. Additionally, deviation from the mean aggregation time is lessened in the presence of KCl.

We used circular dichroism (CD) experiments to probe the secondary structure of αS monomers in the various salts in the presence and absence of KCl, finding no significant effects (Figure S3). Fibril formation in seeded and unseeded conditions was confirmed via Fourier transform infrared (FTIR) spectroscopy and transmission electron microscopy (TEM) (Figure S4). We also found that KCl alone was not sufficient to produce mature αS fibrils (Figure S5).

**Figure 1:**
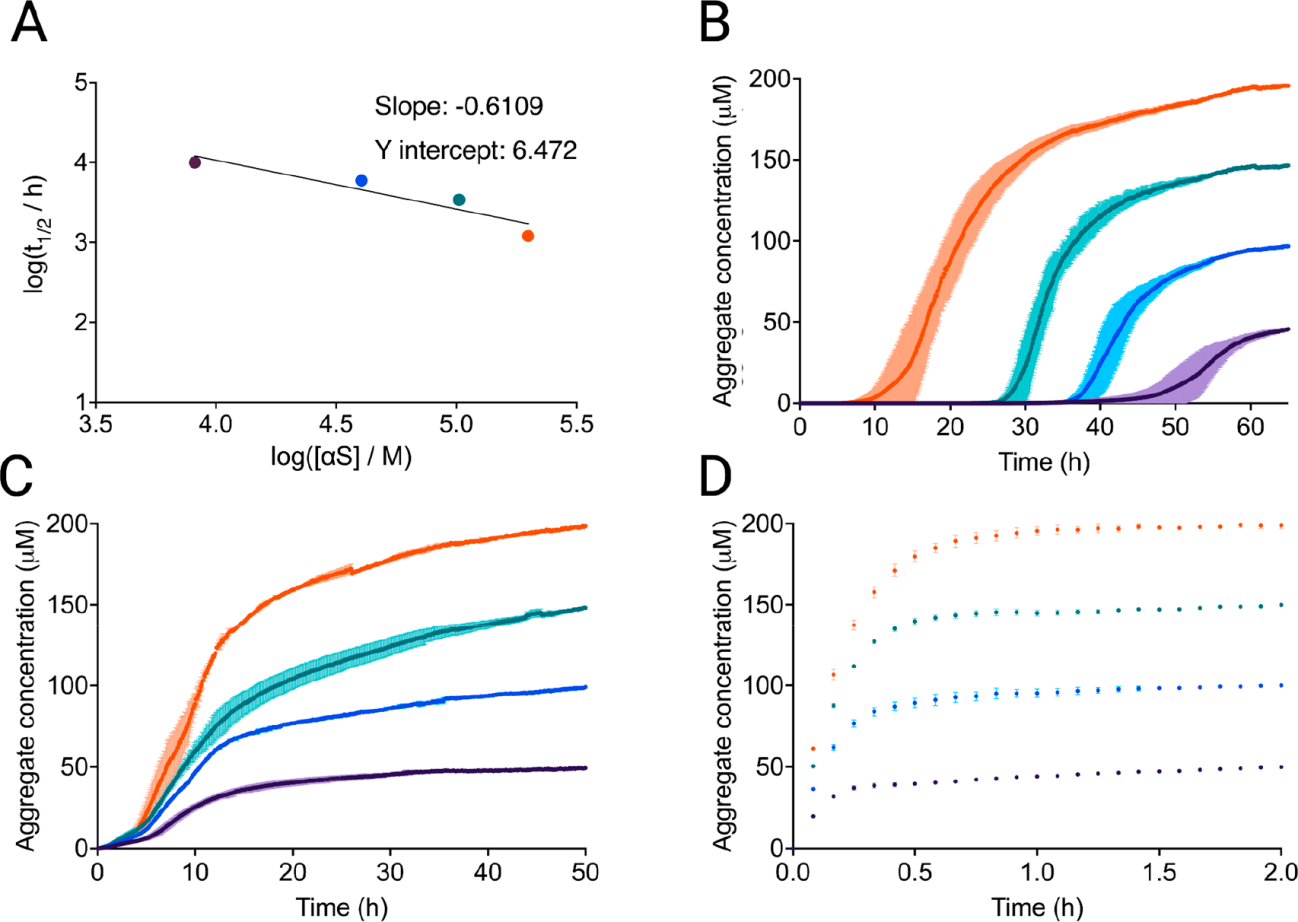
Spontaneous, quiescent aggregation of αS in 150 mM KCl and 400 mM NaPi. αS monomer concentrations are 50 μM (purple), 100 μM (blue), 150 μM (teal), and 200 μM (orange). (A) The scaling exponent, which is the slope of the log–log plot of the monomer concentration (*m*_0_) versus the half-time (*t*_1/2_) for the unseeded aggregation under this condition, is close to −0.5, indicating secondary processes that are independent from monomer concentration within the investigated concentration regime.^35^ (B) Aggregation kinetics of unseeded αS. (C) Aggregation kinetics of αS in the presence of low seed concentration (2.5 nM monomer equivalents). (D) Aggregation kinetics of αS in the presence of high seed concentration (5 μM monomer equivalents). The end points are normalized to the αS monomer concentration at the end of the experiment, which was detected via the Pierce BCA Protein Assay.

With these reaction parameters, an αS aggregation assay could be conducted in 2 days at pH 7.4. The kinetics of the quiescent aggregation of αS were then investigated via serial dilutions of input monomer under various seeding conditions to glean information on the microscopic mechanisms that drive aggregation.^15−17^ We then analyzed the dependence of *t*_1/2_ against the monomer concentration for an unseeded aggregation allowing derivation of a scaling exponent^17^ (Figure 1A). This type of analysis was not previously possible for unseeded αS aggregation. The scaling exponent approached the value of −0.5 indicating a weak dependence of the aggregation rate on monomer concentration.^17^ The ThT traces used to derive the log plot are displayed in Figure 1B. Sigmoidal curves, which are indicative of secondary processes, were observed in quiescent conditions at pH 7.4, in contrast to previous aggregation mechanism investigations of αS, which required lowering the pH below 6.5 to observe clear signs of secondary processes.^20,34^

We then measured the ThT fluorescence over increasing input αS monomer, each seeded with 2.5 nM preformed αS fibrils produced in identical reaction conditions (Figure 1C). The significant increase in aggregation speed at these low seed concentrations as compared to the unseeded reaction further confirms that under these conditions secondary processes dominate the production of new fibrils. The introduction of these small concentrations of seeds significantly reduced the monomer concentration-dependence of the reaction, with *t*_1/2_ at 2.5 nM seed being effectively independent of the monomer concentration. Elongation became the dominant mechanism when large amounts of preformed fibrils were present. We observed saturation of the elongation rates at high seed (5 μM) at different concentrations of αS monomer, analogous to the elongation saturation observed previously^20^ at pH 7.4 (Figure 1D).

### Kinetic Analysis of αS Aggregation in High Salt

**Figure 2:**
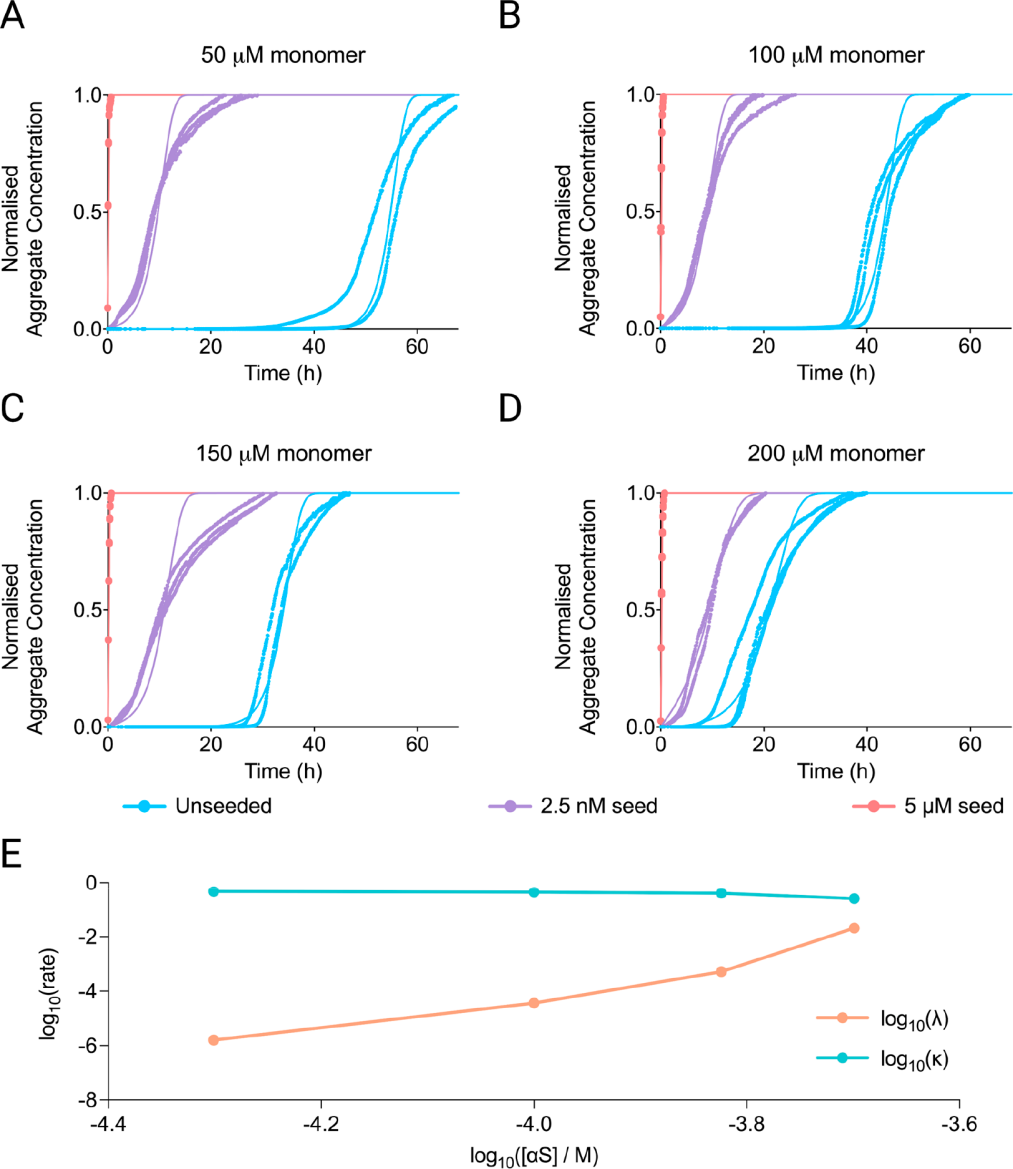
Kinetic analysis of αS aggregation in 150 mM KCl and 400 mM NaPi. (A–D) Fitting of aggregation kinetics with data (points) and fits (solid lines) shown side by side against time (h). Monomer concentrations are 50 μM (A), 100 μM (B), 150 μM (C), and 200 μM (D). (E) Rates of primary (λ) and secondary (κ) processes as a function of monomer concentration are shown on a double logarithmic plot. Secondary processes are essentially independent of the monomer concentration, whereas primary processes are strongly dependent on the monomer concentration. There is also a curvature in the double logarithmic plot of the primary nucleation rate versus the concentration, implying a change in the rate-determining step.^17^

A mechanistic analysis of the data is complicated by the slow approach of the kinetic curves to their plateau value, a feature typical of αS aggregation.^35^ It likely originates from clumping, sedimentation, or other effects not generally modeled explicitly and leads to a deviation of the fitted curves from the experimental ones close to the plateau. To determine the concentration-dependence of the different aggregation steps, we first fitted the sets of seed concentrations at each monomer concentration, extracting the rates of fibril formation via primary (λ) and secondary (κ) processes at each monomer concentration.^13,16^ These fits are shown in Figure 2A–D, while the behavior of λ and κ with varying monomer concentrations is shown in Figure 2E. The fits confirm the strong dominance of secondary processes over primary ones. However, given the low reaction order of the secondary process, it was not possible to determine whether the dominant secondary process was fragmentation or secondary nucleation based on the kinetics alone. This is a well-known effect, as demonstrated previously.^35^ We also observed a very strong dependence of primary nucleation on concentration, suggesting a primary reaction order of approximately 13. Given these observations, we then went on to fit all data globally, with a kinetic model that includes a saturated elongation process, a concentration-independent secondary process, and a primary nucleation process (Figure S6). The model matches well the seed dependence, monomer dependence, and early time and mid time slopes, but fails to reproduce the slow approach to the plateau, overall supporting the above mechanistic conclusions. Particularly noticeable is the high reaction order of primary nucleation, suggesting a large nucleus size, in line with the difficulty of triggering primary nucleation in αS aggregation. We furthermore confirm that the behavior observed here cannot be adequately explained by a model where secondary processes are negligible (Figure S7).

### Small Molecule Inhibitors of Secondary Nucleation in High Salt

**Figure 3:**
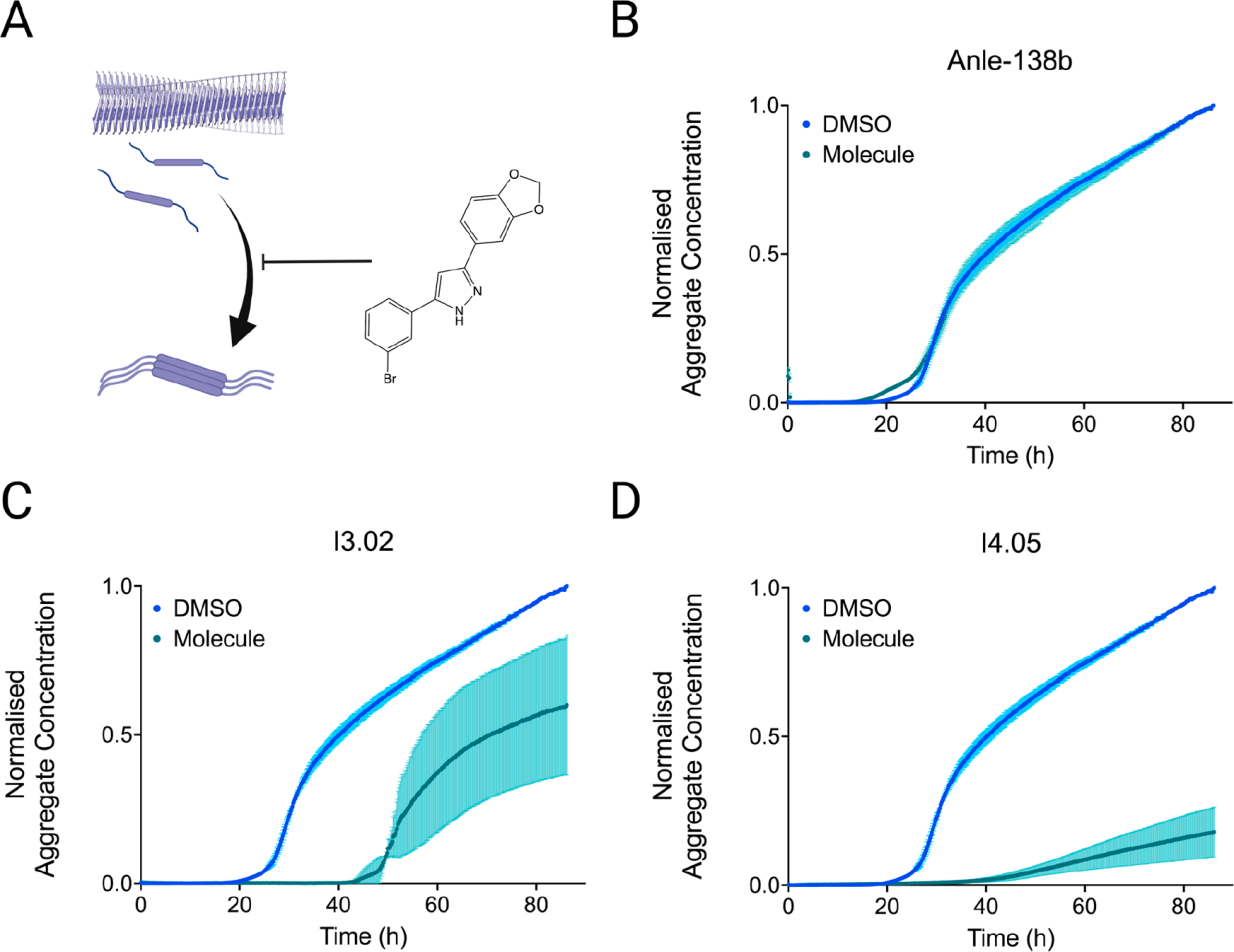
Small molecule inhibitors of secondary nucleation in the aggregation assay reported in this work. (A) Schematic of the aggregation process. A dominant mechanism in oligomer formation is the nucleation of aggregates from the surfaces of the existing ones (secondary nucleation). Small molecules can block this process by blocking the nucleation sites on the surface of the fibrils.^10,36,38^ (B–D) Kinetic traces are shown of a 100 μM solution of αS pH 7.4, 37 °C with 150 mM KCl and 400 mM NaPi in the presence of 1% DMSO alone (light blue) or at 50 μM molecule in 1% DMSO (teal): Anle-138b (B), I3.02 (C), and I4.05 (D).

A set of small molecules that had previously been identified as αS secondary nucleation inhibitors^36^ were also tested here, as a test of both the mechanism of aggregation and of the efficacy of the molecules themselves. The two most potent molecules previously reported, I3.02 and I4.05, showed high levels of inhibition (Figure 3). The fact that the aggregation reaction in the assay reported in this work can be slowed by inhibitors of secondary nucleation suggests that this process, rather than fragmentation, dominates here. For comparison, Anle-138b, a control compound that previously exhibited mild αS aggregation inhibition,^37^ is also shown with little observable inhibition. The plateau drift in the presence of DMSO may have resulted from interactions between the organic solvent and high salt concentration.

## Conclusions

In this work, we have reported an in vitro aggregation assay to study the spontaneous aggregation of αS under quiescent conditions at physiological pH. This assay adds to an increasing repertoire of seeded and unseeded αS assays (Table S1). While modulating the importance of different microscopic processes by changing solution conditions is well established,^20−22,34^ this work provides a framework to observe secondary nucleation processes at neutral pH and greatly accelerates the primary nucleation of αS without the need for surfaces or interfaces. We show that both anionic and cationic species were critical to this optimization, which may function to neutralize side-chain charge along the axis of the growing fibrils. This work is consistent with the finding that structures of αS fibrils from MSA patient brains resolved by cryo-EM revealed unknown electron dense entities packed into the interfilament interface, surrounded by many positively charged residues. This suggested that charged cofactors may be packed into the interfilament interfaces. As a mechanistic test, small molecules previously reported to inhibit secondary nucleation also showed significant efficacy, suggesting that secondary nucleation contributes significantly to the aggregation process under the conditions used here. Although it remains to be seen if the in vitro αS assay reported in this work can faithfully replicate ex vivo fibrils, we suggest that this work can inform drug discovery and mechanistic investigations for PD and related synucleinopathies.^10,36,38^

## Materials and Methods

### Purification of αS

The N-terminal acetylated wild-type αS was purified as described previously.^20−22,34,39^ Briefly, αS was produced by cotransforming *E. coli* with the pT7-7 plasmid and an expression pACYCduet plasmid encoding a yeast N-terminal acetyltransferase (NatB), provided by Dr. Dan Mulvihill, University of Kent, Canterbury, UK.^24^ Recombinant αS was purified as described previously. The plasmid pT7-7 encoding for human αS was transformed into BL21-competent cells alongside an expression pACYCduet plasmid encoding a yeast N-terminal acetyltransferase (NatB), provided by Dr. Dan Mulvihill, University of Kent, Canterbury, UK. Following transformation, competent cells were grown in LB in the presence of ampicillin (100 μg/mL). Cells were induced with IPTG and grown overnight at 37 °C and harvested by centrifugation in a Beckman Avanti J25 centrifuge with a JA-20 rotor at 5000 rpm (Beckman Coulter, Fullerton, CA). The cell pellet was resuspended in 10 mM Tris, pH 8.0, 1 mM EDTA, 1 mM PMSF and lysed by multiple freeze–thaw cycles and sonication. The cell suspension was boiled for 20 min and centrifuged at 13 500 rpm with a JA-20 rotor (Beckman Coulter). Streptomycin sulfate was added to the supernatant to a final concentration of 10 mg/mL, and the mixture was stirred for 15 min at 4 °C. After centrifugation at 13 500 rpm, the supernatant was taken with an addition of 0.36 g/mL ammonium sulfate. The solution was stirred for 30 min at 4 °C and centrifuged again at 13 500 rpm. The pellet was resuspended in 25 mM Tris, pH 7.7, and ion-exchange chromatography was performed using a HQ/M-column of buffer A (25 mM Tris, pH 7.7) and buffer B (25 mM Tris, pH 7.7, 600 mM NaCl). The fractions containing αS (∼300 μM) were dialyzed overnight against the appropriate buffer. Aliquots were flash-frozen in liquid N_2_ and stored at −80 °C. The presence of N-terminal acetylation was verified by mass spectrometry, and the protein concentration was determined spectrophotometrically using ε_275_ = 5600 M^–1^ cm^–1^.

### Aggregation Assays

All aggregation assays were performed on fresh αS following size exclusion chromatography (SEC) through a Superdex 75 Increase 10/300 GL. Additional uses of excess αS from previous experiments followed a freeze–thaw cycle at −20 °C, concentration, and further SEC immediately prior to kinetic analysis to remove the preformed seeds. αS was exchanged by SEC into 20 mM NaPi pH 7.4, after which it was combined with the designated salts for kinetic analysis. All reactions were performed at 100 uL in Corning 3881 96-well half area plates at 37 °C with an aluminum plate sticker to mitigate evaporation. Plates were incubated in FluoStar LITE plate readers (OMEGA) for up to 7 days with periodic ThT fluorescence measurements, in the absence of shaking. Fitting of kinetic models followed the Amylofit recommendations.^17^ Elongation experiments were conducted by adding 15 uM preformed fibrils to a dilution series of αS in 400 mM sodium phosphate, 150 mM KCl, pH 7.4, as above. For inhibition testing, the molecules (or DMSO alone) were then added at the desired concentration to a final DMSO concentration of 1% (v/v).

### Fourier Transform Infrared (FTIR) Spectroscopy

αS fibrils were recovered from completed spontaneous and seeded aggregation assays following plateau of the ThT fluorescence. Fibrils were centrifuged at 21 300 rcf for 10 min before resuspension in water. Samples were dehydrated under light flow of dry air. FTIR spectra were recorded on a Bruker Vertex 70 FTIR spectrometer (Billerica, U.S.) on the diamond ATR, with 4 cm resolution and a data range of 800–4000 cm^–1^; the data in the amide peak 1 (1580–1720 cm^–1^) were analyzed. A rubber band baseline correction was applied to the data, before fitting to a Gaussian equation with 4–7 peaks. The area under each peak was integrated to obtain relative compositions of the secondary structure using the following classifications: peaks under 1640 cm^–1^ were assigned to β-sheet structures, peaks from 1640 to 1660 cm^–1^ were assigned to disordered random coils/α-helices, and peaks above 1660 and 1685 cm^–1^ were also assigned to β-sheet structures.

### Transmission Electron Microscopy (TEM)

αS reaction samples were recovered by pipetting up and down after the ThT plateau as described above. TEM images were obtained with the assistance of the electron microscopy specialist Dr. Heather Greer of the in-house (Department of Chemistry) TEM facility. Copper Quantifoil R2/2 grids (Quantifoil GmbH, Germany) were first glow-discharged before 2.5 μL of the samples was applied for 40 s. The excess sample was carefully absorbed using blotting paper, and then the grids were stained using 1.5 wt % uranyl acetate for 40 s. The excess uranyl acetate was removed using blotting paper. Micrographs were acquired using a Talos F200X G2 electron microscope operating at 200 kV (FEI, Hillsboro, OR). Digital micrographs were acquired on a Ceta 16 M camera with speed enhancement using the EMMENU 4 software package (TVIPS, Munich, Germany). The images were analyzed using ImageJ.

## Supporting Information Available

Initial aggregation experiments with different salts, a summary of previous aggregation conditions for αS, CD experiments investigating salt effects on folding, structural characterization of the αS fibrils via FTIR and TEM, and a global fit of all experiments presented in Figures 1 and 2 (PDF)
