# Supplementary material for: Secondary Processes Dominate the Quiescent, Spontaneous Aggregation of α-Synuclein at Physiological pH with Sodium Salts
Source: ACS Chem Neurosci. 2023 Aug 14;14(17):3125–31. doi: 10.1021/acschemneuro.3c00282 (PMC10485892; doi:10.1021/acschemneuro.3c00282)
Supplement: Supplementary file 1 — cn3c00282_si_001.pdf [file cn3c00282_si_001.pdf]

SUPPORTING INFORMATION

Secondary Processes Dominate the Quiescent, Spontaneous Aggregation of α-Synuclein at Physiological pH with Sodium Salts

Robert I. Horne[1*], Michael A. Metrick II[1,2*], Wing Man[1],
Dillon Rinauro[1], Z. Faidon Brotzakis[1], Sean Chia[1,3], Georg Meisl[1]
and Michele Vendruscolo[1+]

[1]Centre for Misfolding Diseases, Yusuf Hamied Department of Chemistry, University of Cambridge, Cambridge, UK
[2]College of Medicine, University of Illinois at Chicago, Chicago, USA
*[3]Bioprocessing Technology Institute, Agency of Science, Technology and Research (A*STAR), Singapore*

[+] Correspondence to: mv245@cam.ac.uk

Citation	Assay conditions	Kinetic model
This work	Unseeded, 400 mM NaPi, 150 mM KCl, pH 7.4	Spontaneous primary nucleation with dominant secondary processes
Gaspar et al. (2017)[1]	Seeded, 10 mM MES pH 5.5	Secondary nucleation dominated at low seed concentrations
Flagmeier et al. (2016)[2]	Seeded, 20 mM NaPi, pH 4.8	Secondary nucleation dominated at low seed concentrations
Galvagnion et al. (2015)[3]	DMPS lipids, 20 mM NaPi, pH 6.5-7.4	Heterogeneous nucleation, potential secondary character
Buell et al. (2014)[4]	Seeded, 20 mM NaPi, pH 6.5 - 7.4	Elongation dominated

Table S1. Summary of the quiescent αS aggregation assays and their kinetic models mentioned in this work.

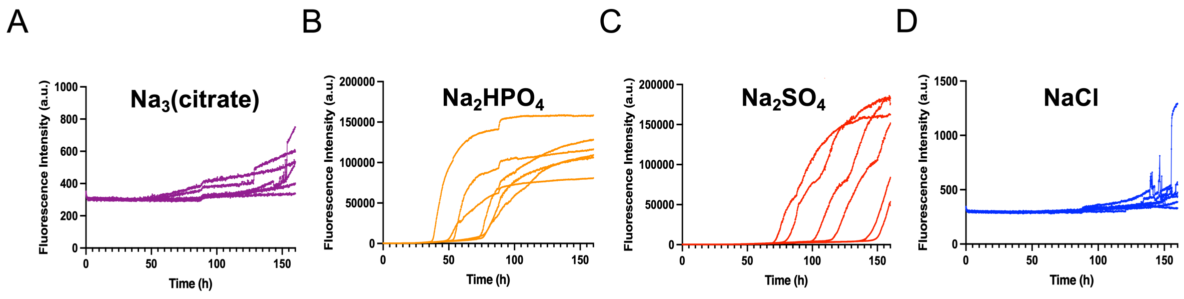

Figure S1. Aggregation of αS in the presence of salts. (A-D) The aggregation of 100 μM αS was monitored in replicates of 6 using ThT fluorescence at pH 7.4 and 37 °C under quiescent conditions. Salts included were: 167 mM Na_3citrate (purple) (**A**), 400 mM Na_2HPO_4 (orange) (**B**), 334 mM Na_2SO_4 (red) (**C**), 1000 mM NaCl (blue) (**D**).

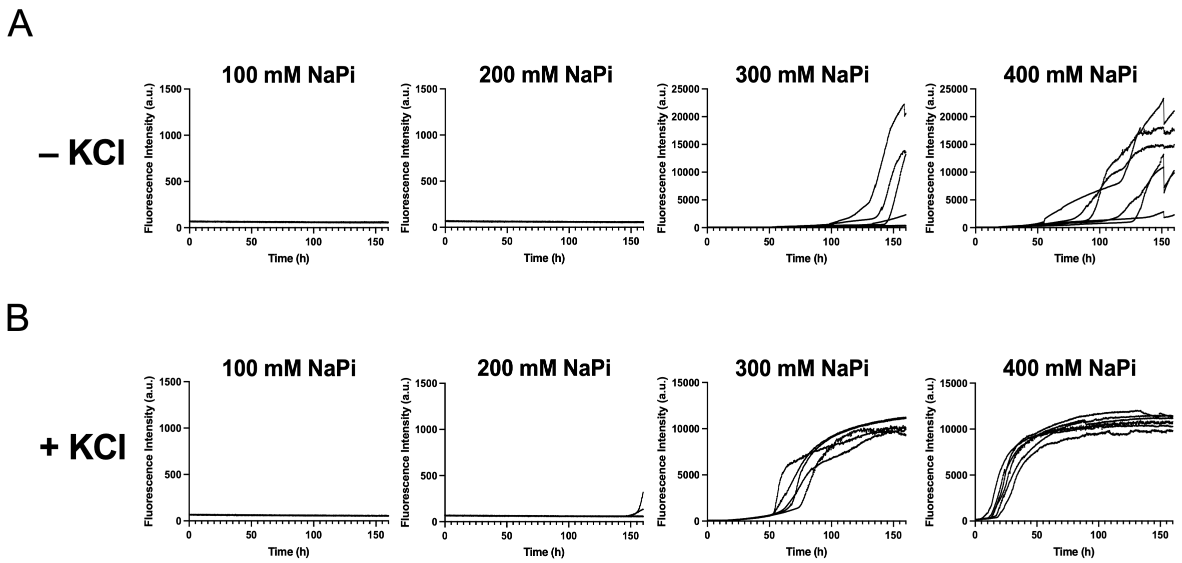

Figure S2. Kinetic traces of αS (100 μM) aggregation in presence and absence of 150 mM KCl with varying concentrations of NaPi. (**A,B**) The aggregation was monitored using ThT fluorescence at pH 7.4, 37 °C under quiescent conditions in the absence (**A**) and presence (**B**) of 150 mM KCl. Different NaPi concentrations (100 mM, 200 mM, 300 mM, and 400 mM) were also screened.

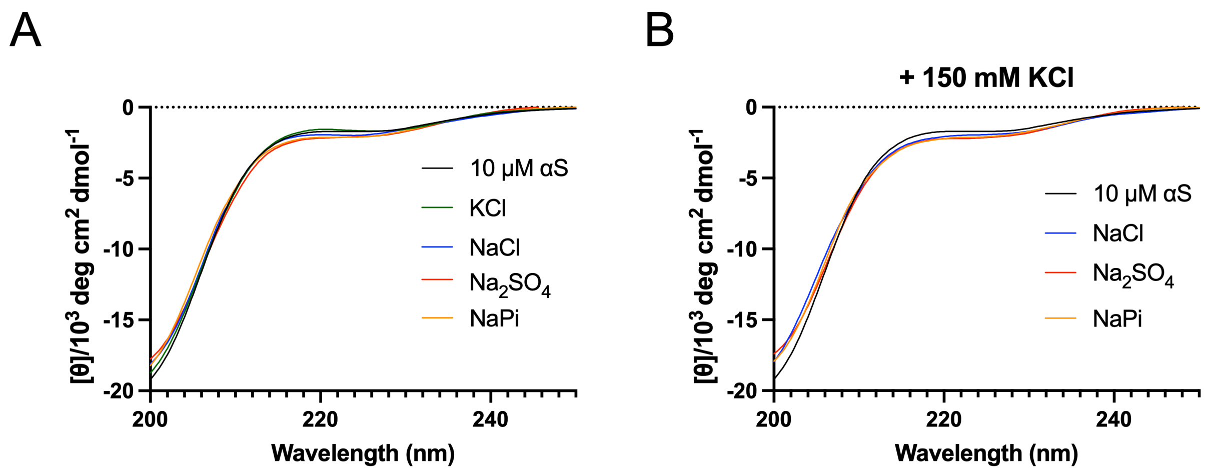

Figure S3. The monomeric structure of αS is only weakly perturbed in presence of anions. (**A,B**) CD measurements are illustrated for αS (10 μM) in the absence of KCl (**A**) and presence of KCl (**B**) with the addition of various anions at 1000 mM ionic strength. The anions included 1000 mM NaCl (blue), 334 mM Na_2SO_4 (red), 400 mM Na_2HPO_4 (yellow). The measurements for Na_3citrate were attempted but the experimental conditions were not compatible with the instrument. All experiments at pH 7.4 and at 10 °C.

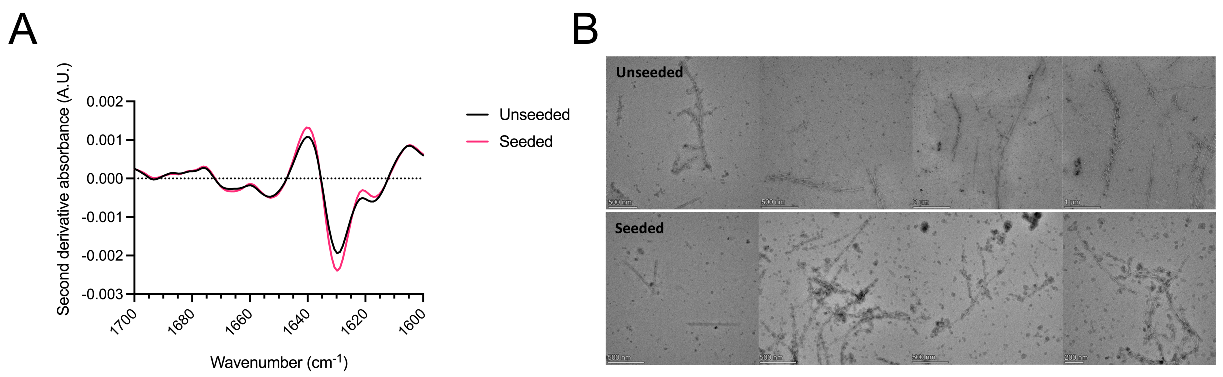

Figure S4. Structural characterization of the αS fibrils generated in the presence of 150 mM KCl and 400 mM NaPi. (**A**) Second derivative FTIR spectra of αS reaction products formed in 400 mM NaPi, 150 mM KCl in the presence (red) and absence (black) of preformed αS. (**B**) TEM morphology of seeded and unseeded αS fibrils. Fibrils were collected from kinetic experiments with conditions of 150 mM KCl and 400 mM NaPi and recorded using TEM. Representing images of unseeded (top) and seeded (bottom) fibrils are shown.

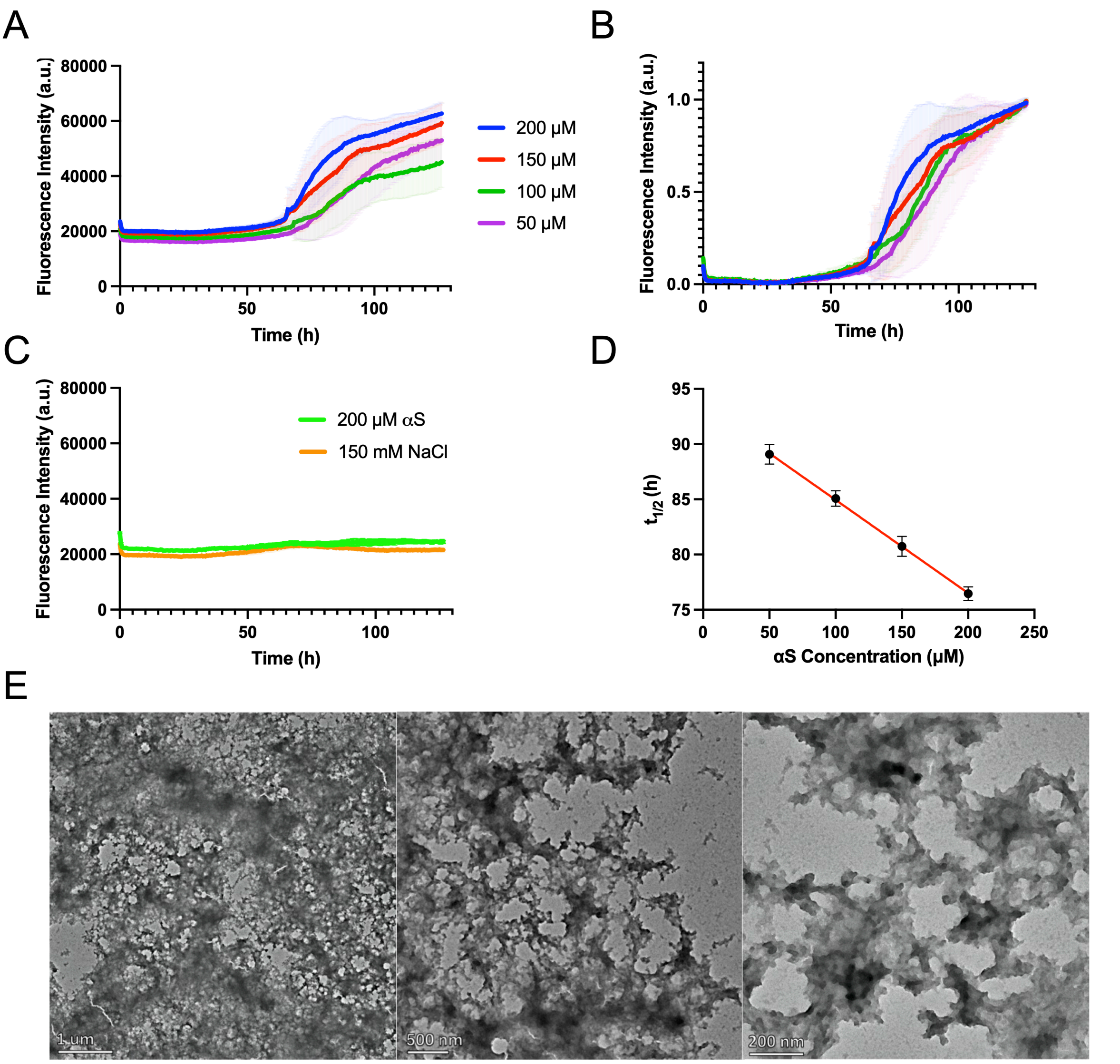

Figure S5. αS forms amorphous aggregates in presence of 150 mM KCl and in the absence of NaPi. The aggregation was monitored using ThT fluorescence in 20 mM NaPi buffer pH 7.4 and 150 mM KCl under quiescent conditions at 37 °C. (**A,B**) Raw kinetic traces (**A**) and normalised kinetic traces (**B**) of 200 µM (blue), 150 µM (red), 100 µM (green) and 50 µM (purple) of αS monomer. (**C**) Controls of 200 µM αS without KCl in the absence (neon green) and presence of NaCl (yellow). The mean experimental replicate for each condition is shown in a solid line with the standard deviation of experimental replicates in error bars. (**D**) A negative correlation is shown for $t_{1/2}$ values vs protein concentration with P-value = 0.003 and R^2 = 0.99. The $t_{1/2}$ values were 89.5 ± 1.2, 84. 7 ± 1.1, 81.6 ± 1.1 and 77.1 ±0.7 for 50, 100, 150 and 200 uM αS, respectively. **(E)** Samples of 150 mM KCl and 200 µM of αS were collected at the end of ThT experiments and imaged using TEM. The respective scale bars are displayed on the bottom left in individual images.

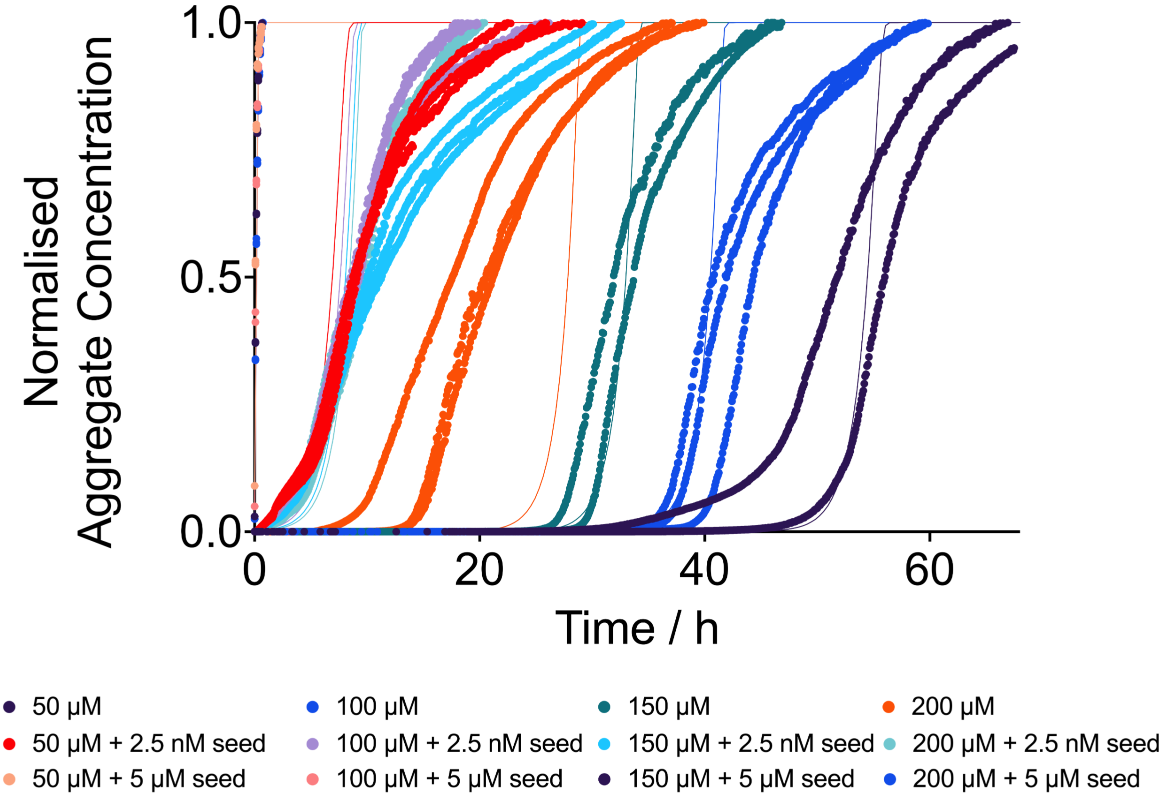

Figure S6. Alternative kinetic analysis of αS aggregation. Global fits of the kinetics at all monomer and seed concentrations to a model including a saturated elongation process, a concentration independent secondary process and a primary nucleation process with reaction order 13. The model matches well the seed dependence, monomer dependence early time and mid time slopes, but fails to reproduce the slow approach to the plateau (a known issue) and misses the highest unseeded concentration. The latter is due to the fact that the model does not include a change in rate-determining step for primary nucleation to account for the increase in concentration dependence of this step between 150 and 200 μM, which can be seen in **Figure 2**. The level to which the global fits match the data confirms the conclusions drawn from the analysis in **Figure 2**.

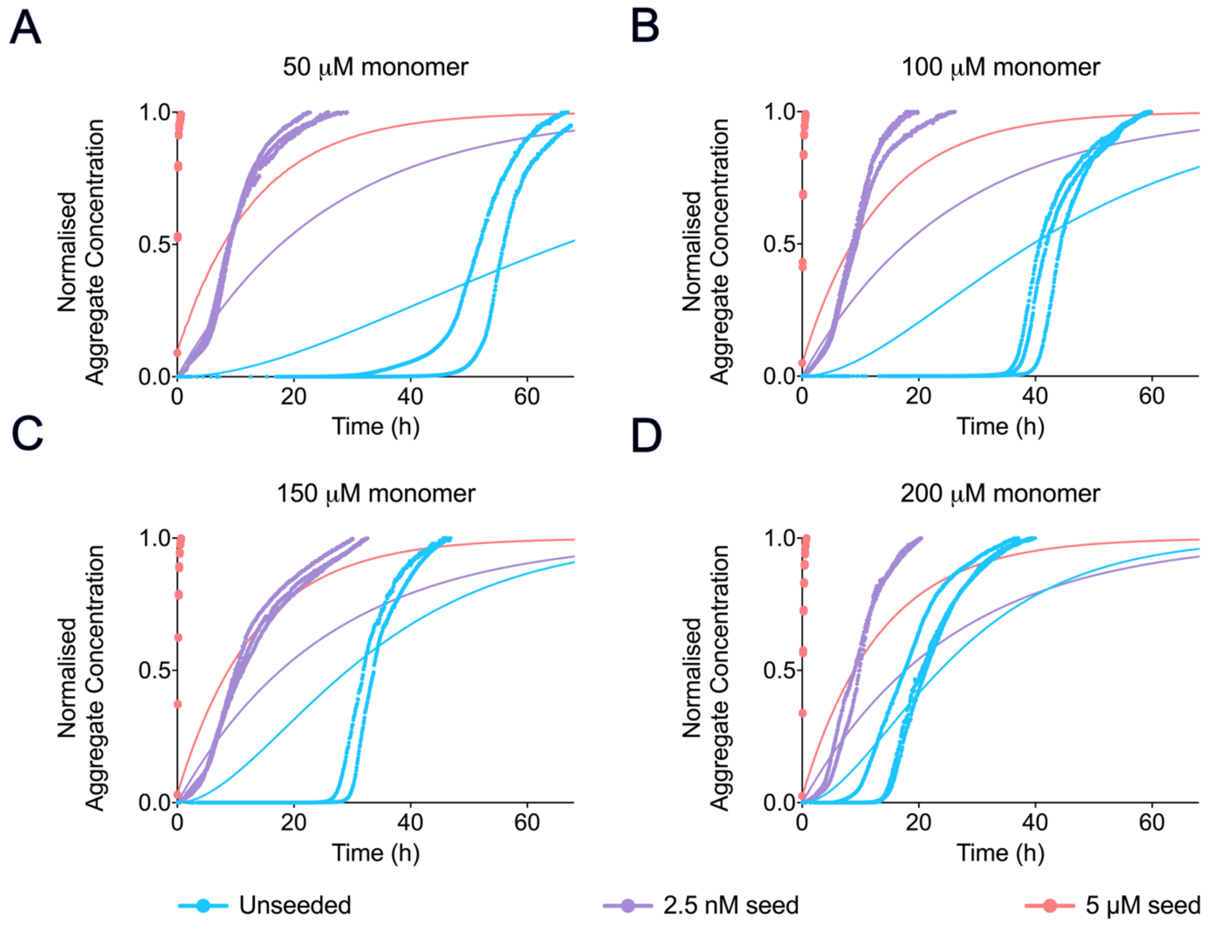

Figure S7. Kinetic analysis of αS aggregation in 150 mM KCl and 400 mM NaPi. (A-D) Fitting of aggregation kinetics with data (points) and fits (solid lines) shown side by side against time (h). Monomer concentrations are: 50 μM (**A**), 100 μM (**B**), 150 μM (**C**), 200 μM (**D**). The combined rate of primary nucleation and elongation is the free parameter for unseeded aggregation, and for the high (5 μM) and low (2.5 nM) seeded aggregation a model is fitted with negligible secondary and primary nucleation, leaving elongation as the only free parameter. Only the high seed data can be reproduced by a model that does not include any nucleation processes, confirming that elongation dominates over nucleation only in this limit.

Supplementary References

1. Gaspar, R., Meisl, G., Buell, A. K., Young, L., Kaminski, C. F., Knowles, T. P., Sparr, E., Linse, S. (2017) Secondary nucleation of monomers on fibril surface dominates α-synuclein aggregation and provides autocatalytic amyloid amplification. *Q. Rev. Bioph. 50*, e6.
2. Flagmeier, P., Meisl, G., Vendruscolo, M., Knowles, T. P., Dobson, C. M., Buell, A. K., Galvagnion, C. (2016) Mutations associated with familial Parkinson's disease alter the initiation and amplification steps of α-synuclein aggregation. *Proc. Natl. Acad. Sci. USA 113* (37), 10328-10333.
3. Galvagnion, C., Buell, A. K., Meisl, G., Michaels, T. C., Vendruscolo, M., Knowles, T. P., Dobson, C. M. (2015) Lipid vesicles trigger α-synuclein aggregation by stimulating primary nucleation. *Nat. Chem. Biol. 11* (3), 229-234.
4. Buell, A. K., Galvagnion, C., Gaspar, R., Sparr, E., Vendruscolo, M., Knowles, T. P., Linse, S., Dobson, C. M. (2014) Solution conditions determine the relative importance of nucleation and growth processes in α-synuclein aggregation. *Proc. Natl. Acad. Sci. USA 111* (21), 7671-7676.
